# Contraceptive Use and Its Associated Factors among Women Who Gave Birth within 12 Months in Dubti Town, Pastoral Community, of Afar Region Northeast, Ethiopia

**DOI:** 10.1155/2021/6617189

**Published:** 2021-07-07

**Authors:** Abdu Yimam, Girmatsion Fisseha, Mebrahtu Kalayu, Etsay Woldu Anbesu

**Affiliations:** ^1^Save the Children, Afar Region, Ethiopia; ^2^School of Public Health, College of Health Science, Mekelle University, Tigray, Ethiopia; ^3^Department of Public Health, College of Medical and Health Sciences, Samara University, Afar, Ethiopia

## Abstract

**Introduction:**

Substantial numbers of women are not using contraceptives in their postpartum period and die due to avoidable causes related to birth complications. Contraceptives use within 12 months of childbirth has given less attention in Ethiopia. Thus, this study is aimed to assess contraceptive use and its associated factors among women who gave birth within 12 months in Dubti town, pastoral community of Afar region, Ethiopia.

**Methods:**

A community-based cross-sectional study was conducted among 342 women in the Dubti town. A systematic random sampling technique was employed to identify and enroll women. Data were collected using a pretested, structured, and interviewer-administered questionnaire. Descriptive statistics were done, and logistic regression analysis was employed to identify the factors associated with contraceptive use. The statistical association was measured by odds ratio with a 95% confidence interval. *p* value < 0.05 was considered as statistically significant.

**Results:**

In this study, 103 (30.1%) [95% CI: 25.4%, 35.1%] women have used contraceptives. Women who had secondary educational level (AOR = 3.53, 95% CI (1.68, 7.36), had antenatal care follow-up (AOR = 1.93, 95% CI (1.01, 3.69), and visited by health worker after delivery (AOR = 2.54, 95% CI (1.37, 4.68) were associated with increased odds of contraceptives use.

**Conclusions:**

This study revealed that the prevalence of contraceptive use was low compared to the national recommended figure. Secondary educational level, having antenatal care follow-up, and being visited by health workers after delivery were predictors of contraceptive use. Thus, increase the educational status of women, antenatal care follow-up service, and visiting after delivery by health workers are important interventions to promote the use of contraceptives in the postpartum period.

## 1. Introduction

Family planning is a means of promoting the health of mothers, families, and reduce high maternal, infant, and child mortality. Postpartum contraceptive is the prevention of unintended and closely spaced pregnancies during the first 12 months after childbirth [1]. Substantial numbers of women are not using contraceptives in their postpartum period, which leads to a risk of pregnancy [2]. A systematic review and meta-analysis of postpartum contraceptive use among women in low- and middle-income countries indicated that the overall pooled modern contraceptive prevalence was 41.2% and the lowest in West Africa (36.3%). The pooled prevalence of unmet need was 48.5% and the highest in South Asia/South East Asia (59.4%) [2]. Ethiopia has the lowest postpartum care coverage of 13% compared to Sub-Saharan Africa country, and the Afar region has 6.5% postpartum care coverage which is the lowest compared to other regions of Ethiopia. Moreover, the national contraceptive coverage is low among currently married women 35% and, in the Afar region, has the lowest 12% compared to the national figure [3].

Pregnancies occurring within a year of the mother's previous birth are riskier for the health of both the mother and the child, and these increase childhood and maternal mortality [4, 5]. Contraceptives use can avert more than 30% of maternal deaths and 10% of child mortality [6].

Review of literature showed that age of mothers, religion, ethnicity, marital status, birth interval, family size, parental education, parental occupation, household wealth index, ever used contraceptive, the number of children, history of abortion, menses resumed, started sex, planned birth, antenatal care follow-up, place of delivery, postnatal care, knowledge, and attitude on contraceptive use were factors affecting contraceptives use [7–16].

In Ethiopia, though the World Health Organization (WHO) and Ethiopian family planning program recommend an interval of at least 2 years following a live birth [17, 18], still fertility is high about 5.8 particularly in pastoral regions [3]. Moreover, though improvement in increased contraceptive utilization coverage and reduction of maternal mortality 412 per 100,000 live birth due to strategies implemented by government and nongovernmental organizations [3], women still die due to avoidable causes related to pregnancy and birth complications. Contraceptives use during postpartum the period has given less attention and no indicator measurement in Ethiopia's health management information system. Thus, this study aimed to assess contraceptive use and its associated factors among women who gave birth within 12 months in Dubti town, pastoral community of Afar region, Northeast Ethiopia in 2018.

## 2. Methods

### 2.1. Study Setting and Period

Dubti town is located in Dubti district which is one of the eight districts of Zone one. It is 595 km northeast of Addis Ababa. Dubti town has 1 kebele (the lowest administrative structure of district) which has 38 ketenas (the lowest sub administrative structure of kebele). Based on the 2007 Census population projection, the number of women in the reproductive age in Dubti town was 2586, and the number of women within the last 12 months of childbirth was 1091. Dubti town has 1 health center and one general hospital.

### 2.2. Study Design and Population

A community-based cross-sectional study was conducted among all systematically selected women who give birth within the last 12 months in Dubti town. The women, who were ill during the study period and were unable to respond, were excluded from the study.

### 2.3. Sample Size Determination and Sampling Techniques

The sample size was determined by using the single population proportion formula with the following assumptions: considering 5% type one error, 95% confidence interval (CI), and 29.3% proportion of contraceptive use within 12 months of childbirth [19]. Then, we added 10% nonresponse, and the final sample size was 350 participants.

Out of thirty-eight ketenas, 10 ketenas were selected by using the lottery method. The calculated sample size was proportionally allocated to randomly selected ketenas. There was a total of 1091 women delivered within the last 12 months prior to data collection period in the randomly selected ketenas (i.e., Ketena − 2 = 80, Ketena − 4 = 120, Ketena − 7 = 95, Ketena − 9 = 115, ktena − 11 = 106, ketene − 15 = 75, ketene − 22 = 125, ketene − 24 = 121, Ketena − 30 = 109, ketene − 32 = 145). A systematic random sampling technique was employed to enroll the study participants. Accordingly, every three participants were selected by using a systematic random sampling technique till the required sample size has been reached.

### 2.4. Study Variables

The outcome variable of this study was contraceptive use. It was defined as if mothers used contraceptives to prevent unintended and closely spaced pregnancies within the first 12 months period following birth. It was coded as “1” for “yes” response while “0” for “no” response during analysis.

The independent variables were sociodemographic characteristics (age of mothers, religion, ethnicity, marital status, birth interval, family size, parental education, parental occupation, and household income), reproductive and health services related characteristics (ever used contraceptive, number of children, history of abortion, menses resumed, started sex, planned birth, current pregnancy status, antenatal care follow-up, place of delivery, and postnatal care), and knowledge and attitude on contraceptive use. Good knowledge was measured if mothers scored 75%-100% of the total knowledge-related questions unless considered as having poor knowledge (scoring of <75%). The attitude of mothers to words contraceptives was measured by asking mothers about their subjective opinion, outlook, position, and ideas towards contraceptive methods. Mothers were classified as having a positive attitude if scored 75-100% from total attitude questions and considered as having a negative attitude if scored ≤75% attitude questions [16].

### 2.5. Data Collection Tools and Techniques

Data were collected by using a pretested, structured, and interviewer-administered questionnaire adapted from literature reviews and the Ethiopian Demographic and Health Survey [3]. The adapted questionnaire was modified to fit the local circumstances and the research objective. The questionnaire was prepared first in English and translated into Amharic by an English instructor who is fluent in both languages. The Amharic questioner was used to collect the data. The tool was pretested on 18 women other than the source population in the Samar-logia city administration. The pretest was done to ensure clarity, wordings, logical sequence, and skip patterns of the questions. Then, amendments to the questionnaire were made accordingly. The data were collected by six preparatory completed students and supervised by two diploma nurses. The supervisors checked the day-to-day activities of data collectors regarding the completion of questionnaires, clarity, and proper coding of the responses.

### 2.6. Data Quality Control

Students who can speak the local language were recruited as data collectors. The data collectors and the supervisors were trained for one and half days by the principal investigator on the study tool, objective, consent form, and data collection procedure.

### 2.7. Data Management and Analysis

The data were checked for completeness, coded, and entered into the Epi info software, and exported to SPSS version 20 for analysis. The descriptive analysis was done, and the results were presented using texts, frequency tables, figures, and mean with standard deviation.

A binary logistic regression analysis was done to assess the association between the outcome variable with each explanatory variable. Independent variables with a *p* value less than 0.25 in the bivariable logistic regression analysis were considered for the final model. Correlation between independent variables was assessed but we did not find any correlation. Finally, multivariable logistic regression analysis was done to control potential confounders and to identify the factors associated with the outcome variable. A statistical significance level was declared at a *p* value of less than 0.05.

### 2.8. Ethical Consideration

The study was approved by the Samara University Research and Ethical Review Committee (RERC) on 18, November 2017, with RE ERC 0029/2017. An official letter was written from the Samara University to Dubti District Administration Office. Then, permission and support letter was written by Dubti urban kebele. The women enrolled in the study were informed about the study objectives, benefits, and risks associated with it. Verbal consent was taken from the women before the interview. Confidentiality of responses was maintained throughout the study. For teenagers <, 18 years consent form was signed by their husbands.

## 3. Result and Discussion

### 3.1. Sociodemographic Characteristics of Participants

In this study, a total of 342 women have included the study with a response rate of 97.8%. The mean age of women was 29.82 years (with a standard deviation of ±7.45) and about 213 (62.3%) of the mothers were in the age group of 21–34 years. 236 (69%) women were Muslim, and 156 (45.6%) were Afar in the Ethnic. About 106 (31%) were illiterate, and 175 (51.2%) women gave their last birth within birth interval <24 months (Table 1).

### 3.2. Reproductive and Health Services Related Characteristics

In this study, 117 (34.2%) women have ever used contraceptives, 135 (39.5%) had no planned birth, 233 (68.1%) had ANC follow-up, 25 (7.3%) were pregnant, 135 (39.5%) delivered at home, and 82 (24%) were visited by health workers after delivery. 103 (30.1%) [95% CI: 25.4%, 35.1%] women have used contraceptives. Of this, 68 (66%) of them used injectable. 70 (67.9%) women have used contraceptives from private health facilities. The main perceived reason not to use contraceptives was exclusive breastfeeding 154 (64.4%) (Table 2).

### 3.3. Knowledge and Attitude of the Study Participants on Contraceptive Use

In this study, 322 (94.2%) women have mentioned the methods of contraceptives, and 275 (85.4%) reported that the sources of information were received from health workers. 329 (96.2%) perceived the use of contraceptives, 110 (38.5%) discussed on contraceptive methods with their husbands, and 79 (71.8%) husbands had a favorable attitude on contraceptives (Table 3).

### 3.4. Factors Associated with Contraceptive Use

The selected variables were tested for their contribution to contraceptive utilization using binary logistic analysis. The variables that showed associations were being secondary educational level, had ANC follow-up services, delivered at a health facility, resumed menses after last delivery, and visited by health worker after delivery. Then, these variables were entered together to determine their effect on the outcome variable on multivariable logistic regression analysis. Accordingly, secondary educational l level (AOR = 3.53, 95% CI (1.68, 7.36), having ANC follow up services (AOR = 1.93, 95% CI (1.01, 3.69) and being visited by health worker after delivery (AOR = 2.54, 95% CI (1.37, 4.68), were factors statistical associated with postpartum contraceptives use (Table 4).

In this study, the odds of contraceptive use within 12 months of childbirth among women who had a secondary educational level were 3.53 times more compared to women who had no educational level (AOR = 3.53, 95% CI (1.68, 7.36). Women who had antenatal care follow-up were 93% more likely to contraceptive use within 12 months of childbirth than their counterparts (AOR = 1.93, 95% CI (1.01, 3.69). The odds of contraceptive use within 12 months of childbirth among women who were visited by health workers after delivery were 2.54 times more compared to their counterparts (AOR = 2.54, 95% CI (1.37, 4.68) (Table 4).

## 4. Discussion

This study was aimed to assess contraceptive use and its associated factors among women who gave birth within 12 months. Based on this, we found that the prevalence of contraceptives use was 30.1% (95% CI, 25.4 to 35.1).

The prevalence of this study was higher than the study done in Kebrebeya, Somali region, Ethiopia region, 12.3% [12], Debat district, Northwest Ethiopia, 10.3% [11]. However, the finding of this study was lower than study conducted in rural Tigray, Ethiopia, 38.3% [20]; Tigray Axum, Ethiopia, 48% [21]; Gonder, 48.4% [7]; Kenya, 49% [22]; Nigerian, 65.6% [23]; Malawi, 75% [8]; and Turkey, 73.7% [24]. This variation might be due to differences in sociodemographic, cultural, and religious, health service accessibility, awareness, and quality differences for the uptake of contraceptive use. Moreover, it might be also due to the temporal difference in the study periods.

In this study, the odds of contraceptive use within 12 months of childbirth among women who had a secondary educational level were 3.53 times more compared to women who had no educational level (AOR = 3.53, 95% CI (1.68, 7.36). This is consistent with a study conducted in Kebribeyah, Ethiopia Somali region [12], Uganda [13], Malawi [8], other settings of developing countries [6], and the USA [9]. This might be because women's attainment of educational level benefits to a better understanding of the available methods of contraceptives during 12 months of childbirth. Besides, educations increase the level of awareness on contraceptives use and to utilize the method of contraceptives. It has been noted that women's education is an important determinant factor for increasing contraceptive use within 12 months of childbirth [13].

Women who had antenatal care follow-up were 93% more likely to contraceptive use within 12 months of childbirth than their counterparts (AOR = 1.93, 95% CI (1.01, 3.69). This is consistent with the study done in Kebribeyah, Somali region, Ethiopia [12]; Gonder town, Northwest Ethiopia [7]; Kenya and Zambia [10]; and the United States [15]. This might be the fact that women who attend antenatal care are more likely to get information on contraceptive use. Moreover, women who had frequent visits during ANC had more exposure to counseling and awareness on birth spacing. A previous study showed that providing education on contraceptive use during antenatal care follow-up had Positive results and women that do not attend ANC are at risk of having closely spaced pregnancies and births [9].

The odds of contraceptive use within 12 months of childbirth among women who were visited by health workers after delivery were 2.54 times more compared to their counterparts (AOR = 2.54, 95% CI (1.37, 4.68) (Table 4). This finding is in line with the study conducted in Kebribeyah, Somali, Ethiopia [12]; Gondar, Ethiopia [7]; Tigray Ethiopia [20]; and United States [15, 16]. This might be the fact that women visited by health workers after delivery can get postnatal care and counseling service on contraceptive use. It has been noted that postpartum educational and counseling services were a significant contribution to contraceptive use within 12 months of childbirth [25, 26].

### 4.1. Strength and Limitation of This Study

The strength of the study was being community-based, and it can represent the source population. However, the study might have a limitation like respondents' recall bias, and as it is, a cross-sectional study design no causal inferences can be made.

## 5. Conclusion

This study revealed that the prevalence of contraceptive use among women who gave birth within 12 months was low compared to the national figure. A secondary educational level, having antenatal care follow-up, and being visited by health workers after delivery were predictors of contraceptive use. Thus, increasing the educational status of women, antenatal care follow-up service, and the visit after delivery by health workers are the important interventions to promote the use of contraceptives in the postpartum period.

## Figures and Tables

**Table 1:** Sociodemographic characteristics of the study participants in Dubti town, pastoral community, Afar region, Northeast Ethiopia, May 2018.

Variables	Category	Frequency (*N*)	Percentage (%)
Age of mothers in years	>19	24	7
	20-34	213	62.3
	>34	105	30.7

Mean age (±SD)	29.82 (±7.45)		

Birth interval in month	No previous birth	48	14
	<24 months	175	51.2
	≥24 months	119	34.8

Religion	Muslim	236	69
	Orthodox	84	24.6
	Protestant	22	6.4

Ethnicity	Afar	156	45.6
	Amhara	124	36.3
	Tigray	49	14.3
	Others (Wolayta, Oromo)	13	3.8

Marital status	Married	286	83.6
	Divorced	29	8.5
	Single	27	7.9

Maternal education	No education	106	31
	Primary	124	36.3
	Secondary	74	21.6
	Higher	38	11.1

Maternal occupation	Housewife	38	11.1
	Pastoral	138	40.4
	Daily labor	36	10.5
	Government	69	20.2
	Small trade	38	11.1
	Students	23	6.7

Husband occupation	Farmer	27	9.5
	Pastoral	105	36.7
	Daily labor	43	15
	Merchant	55	19.2
	Government	56	19.6

Husband education	No education	40	14
	Primary	82	28.7
	Secondary	109	38.1
	Higher	55	19.2

Family size	≤2	74	21.6
	3-4	108	31.6
	5-6	78	22.8
	>6	82	24

Household monthly income (ETBr)	≤500	61	17.8
	501-1000	69	20.2
	>1000	212	62

**Table 2:** Reproductive and health services characteristics of the study participants in Dubti town, pastoral community, Afar region, Northeast Ethiopia, May 2018.

Variables	Category	Frequency (*N*)	Percentage (%)
Number of children	1	48	14
	2-3	170	49.7
	>3	124	36.3

History of abortion	Yes	71	20.8
	No	271	79.2

Number of abortion	1	50	70.4
	2-3	21	29.6

Menses resumed after the last delivery	Yes	287	83.9
	No	55	16.1

Started sex after the last delivery	Yes	299	87.4
	No	43	12.6

Birth planned	Yes	207	60.5
	No	135	39.5

Ever used contraceptive	Yes	117	34.2
	No	225	65.8

Currently using contraceptive	Yes	103	30.1
	No	239	69.9

Types of contraceptive	Pills	29	28.5
	Injectable	68	66
	Implant	6	5.8

Source of the services	Government	33	32.1
	Private	70	67.9

Intension to use contraceptive in the future	Yes	157	65.7
	No	82	34.7

Reason not to use contraceptives	Lack of knowledge	12	5
	Infrequent sex	15	6.3
	Opposition to use	24	10
	Side effect	34	14.2
	Exclusive breastfeeding	154	64.4

Currently pregnant	Yes	25	7.3
	No	317	92.7

Had ANC follow-up	Yes	233	68.1
	No	109	31.9

Number of ANC visits	1-3	168	72.1
	≥4	65	27.9

Place of delivery	Home	135	39.5
	Health facility	207	60.5

Visited by health worker after delivery	Yes	82	24
	No	260	76

**Table 3:** Knowledge and attitude of the study participants on contraceptive use in Dubti town, pastoral community, Afar region, Northeast Ethiopia, May 2018.

Variables	Category	Frequency (*N*)	Percentage (%)
Mentioned methods of contraceptives	Yes	322	94.2

Used after delivery	No	20	5.8

Types of contraceptives ever know (multiple responses)	Pills	321	99.7
	Injectable	263	81.7
	IUCD	258	80.1
	Implant	257	79.8
	Emergency	203	63
	Others (condom)	156	48.4

Had information on contraceptives	Yes	295	91.6
	No	27	8.4

Source of information (multiple responses)	Health workers	275	85.4
	Radio	115	35.7
	TV	161	50
	Friends	186	57.8

Discussed contraceptive methods with husband	Yes	110	38.5
	No	176	61.5

Husband's attitude on contraceptives	Favorable	79	71.8
	Unfavorable	31	28.2

Perceived use of contraceptives	Yes	329	96.2
	No	13	3.8

**Table 4:** Binary and multivariable logistic regression analysis showing factors associated with contraceptive use among study participants in Dubti town, pastoral community, Afar region, Northeast Ethiopia, May 2018.

Variables	Category	Contraceptive use		COR 95% CI	AOR 95% CI
		Yes, *n* (%)	No, *n* (%)		
Maternal education	No education	20 (19.5)	86 (36)	1	1
	Primary	33 (32)	91 (38)	1.56 (0.83, 8.37)	1.1 (0.53, 2.14)
	Secondary	37 (35.9)	37 (15.5)	4.3 (2.21, 8.37)	3.53 (1.68, 7.36)^∗^
	Higher	13 (12.6)	25 (10.5)	2.24 (0.97, 5.12)	1.42 (0.57, 3.53)

History of abortion	Yes	26 (25.2)	45 (18.8)	1.46 (0.84, 2.52)	1.35 (0.67, 2.73)
	No	77(74.8)	194(81.2)	1	1

ANC follow-up	Yes	86 (83.5)	147 (61.5)	3.17 (1.76, 5.7)	1.93 (1.01, 3.69)^∗^
	No	17.6 (16.5)	92 (38.5)	1	1

Place of delivery	Home	31 (30.1)	104 (43.5)	1	1
	Health facilities	72 (69.9)	135 (56.5)	1.78 (1.09, 2.93)	1.24 (0.7, 2.2)

Menses resumed	Yes	93 (90.3)	194 (81.2)	2.16 (1.04, 4.46)	2.2 (0.91, 5.32)
	No	10 (9.7)	45 (18.8)	1	1

Visited by health worker after delivery	Yes	40 (38.8)	42 (17.6)	2.98 (1.77, 4.99)	2.54 (1.37, 4.68)^∗^
	No	63 (61.2)	197 (82.4)	1	1

Ever used contraceptives	Yes	40 (38.8)	77 (32.2)	1.34 (0.83, 2.26)	1.76 (0.99, 3.11)
	No	63 (61.2)	162 (67.8)	1	1

Had information on contraceptives	Yes	92 (95.8)	203 (89.8)	2.61 (0.87, 7.75)	1.76 (0.52. 5.91)
	No	4 (4.2)	23 (10.2)	1	1

^∗^significant at *p* < 0.05.

## Data Availability

The datasets supporting the conclusions of the study are included in the article, and the datasets used for analysis during the current study are available from the corresponding author on reasonable request.
